# Effect of biphasic in vitro maturation (CAPA-IVM) on EGF receptor and embryo development of prepubertal goat oocytes according to follicle size

**DOI:** 10.1080/10495398.2024.2422316

**Published:** 2024-11-10

**Authors:** Mònica Ferrer-Roda, Ana Gil, Maria-Teresa Paramio, Dolors Izquierdo

**Affiliations:** Department of Animal and Food Science, Autonomous University of Barcelona, Barcelona, Spain

**Keywords:** GV chromatin configuration, oocyte competence, follicle diameter, EGFR, epidermal growth factor, blastocyst production

## Abstract

Oocytes spontaneously resume meiosis following their liberation from follicles, preventing full competence acquisition. Biphasic IVM (CAPA-IVM) maintains oocytes in meiotic arrest to improve developmental competence, and it specially affects poorly developed oocytes. We assessed the effect of CAPA-IVM on oocytes from small (<3mm) and large (>3mm) follicles of prepubertal goats. Oocytes were cultured for 6h in pre-IVM with C-type natriuretic peptide (CNP) and estradiol as meiotic inhibitors, and germinal vesicle (GV) rate and chromatin configuration were assessed. Then, oocytes were cultured in conventional IVM (c-IVM) or CAPA-IVM (pre-IVM + c-IVM) and EGF receptor (EGFR) protein expression, intra-oocyte ROS and blastocyst development were assessed. GV rate was higher in CNP groups than control (69% vs 28%, and 67% vs 31%, small and large follicles, respectively), but GV chromatin configuration was similar. In large follicles, EGFR expression was higher in oocytes and cumulus cells after CAPA-IVM, and ROS levels were lower. In small follicles these differences were not observed. c-IVM and CAPA-IVM produced similar blastocyst rates in small (3.7% vs 2.6%, respectively) and large follicles (8.3% vs 2.5%). Overall, CAPA-IVM enhanced EGFR expression for EGF peptide signalling and antioxidant capacity in oocytes from large follicles but oocytes from small follicles were too immature to benefit from it.

## Introduction

Oocyte in vitro maturation (IVM) is a limiting step for in vitro embryo production (IVEP) programs. The oocyte competence for embryo development is reached progressively during folliculogenesis through communication between the oocyte and its somatic cells.^1^ Cumulus cells deliver metabolites and regulatory factors to the oocyte, such as the cyclic nucleotides 3′,5′-cyclic adenosine monophosphate (cAMP) and 3′,5′-cyclic guanine monophosphate (cGMP) to maintain meiotic arrest, and mRNAs and proteins that will sustain early embryo development prior to embryonic genome activation.^2^ This process is mediated by gap junctions communicating the tip of cumulus cells transzonal projections with the ooplasm. After resuming meiosis these projections are withdrawn, and cumulus cells supply to the oocyte is lost.

In livestock species, cumulus-oocyte complexes (COCs) are recovered mostly from small and medium follicles. These oocytes spontaneously resume meiosis in vitro following their liberation from follicles, which prevents the oocyte from fulfilling this process of competence acquisition. Gilchrist et al.^2^ reviewed the use of a pre-maturation (pre-IVM) culture strategy aiming to sustain meiotic arrest while improving cytoplasmic maturation for further synchronization of oocyte nuclear and cytoplasmic maturation, first reported by Franciosi et al.^3^ This biphasic system has been named as CAPA-IVM, which involve a two-step culture: a pre-IVM (or ‘capacitation’—CAPA—culture) in the presence of C-type natriuretic peptide (CNP) as an inhibitor of cAMP degradation, followed by a conventional IVM protocol to induce maturation. As reviewed by Gilchrist et al.,^4^ pre-IVM maintains cumulus-oocyte communication by preserving transzonal projections and open gap junctions, which affects many COC functions linked to developmental competence, such as: (a) germinal vesicle (GV) chromatin condensation to cease transcription in preparation for meiotic resumption; (b) modification of COC metabolism inducing oocyte mitochondrial function and production of antioxidants like glutathione (GSH) to reduce reactive oxygen species (ROS)-induced oxidative stress; and (c) development of the epidermal growth factor (EGF) signalling network to become responsive to EGF-like peptides during IVM. During mammal folliculogenesis, chromatin in the GV is initially decondensed and progressively condenses as the oocyte growths.^5^ This process of large-scale chromatin remodelling is vital to endow meiotic and developmental competence of oocytes.^6^^,^^7^ The EGF network induces cumulus expansion and oocyte nuclear and cytoplasmic maturation after the LH ovulatory surge, but less competent COCs fail to respond to EGF during IVM because of the absence of its receptor (EGFR).^8^^,^^9^ EGFR gene expression has been identified as a potential marker of oocyte competence in bovine cumulus cells at 6h of IVM culture.^10^ The EGFR also contributes to the oocyte cytoplasmic maturation by promoting oocyte mRNA translation,^11^^,^^12^ which is key provided that the oocyte is transcriptionally silent following GVBD and relies on the translation of stored mRNAs for maturation and embryo development. Thus, an increased EGFR gene expression in cumulus cells at the end of IVM was positively related with blastocyst development.^13^ Richani and Gilchrist^14^ reviewed that oocytes are not directly responsive to EGF-like peptides. However, according to our previous results, caprine denuded oocytes contain EGFR protein and its expression is positively related to their follicle size, just like the EGFR in cumulus cells.^15^ Other studies in the goat suggest that EGFR expression in the oocyte is related with oocyte meiotic competence, as gene expression increased after IVM^16^ and protein abundance was higher in meiotically competent oocytes (> 3 mm follicles) compared to incompetent ones (< 0.5 mm follicles).^17^

We have tested the CAPA-IVM protocol with prepubertal goat oocytes, and our results showed that 200 nM of CNP and 10 nM of estradiol in the pre-IVM medium maintained oocytes at the GV stage for 6 hours, sustained cumulus-oocyte communication through the maintenance of transzonal projections, and improved blastocyst development compared to our conventional IVM.^18^ In our study, pre-IVM with CNP delayed GV breakdown (GVBD) when combined with estradiol, related to estradiol promoting expression of CNP receptor NPR2 in cumulus cells.^19^ We did not test the effect of this CAPA-IVM protocol on oocytes from follicles of different sizes. In our laboratory, the blastocyst development of follicles smaller than 3 mm in prepubertal goats was lower compared to those from adult goats, but these differences disappeared if oocytes were recovered from follicles larger than 3 mm of prepubertal goats.^20^ However, in prepubertal goats we obtained only 1.1 follicles larger than 3 mm per ovary.^21^ CAPA-IVM has been suggested to be of more benefit to growing oocytes from small follicles because they benefit from the extra culture time to complete development, whereas it has less impact on fully grown oocytes or on oocytes from larger follicles.^22^

Our objective was to assess the effect of CAPA-IVM on oocyte competence of prepubertal goat oocytes derived from small (< 3 mm) and large (> 3 mm) follicles.

## Materials and methods

Unless otherwise specified, all chemicals and reagents were purchased from Sigma-Aldrich Corporation (St. Louis, USA).

### Oocyte collection

Ovaries from prepubertal and adult goats were retrieved at a local abattoir and transported within 2 h to the laboratory at 35 °C in PBS. Prepubertal goats were 1–2 months old, and adult goats were at the end of their productive life. Only healthy females and morphologically good ovaries were selected.

COCs were recovered according to their follicular diameter. Those from large follicles (≥ 3 mm) were aspirated with a 20 G needle, using the bevel of the needle, which measured exactly 3 mm, as measurement reference; and the others from small follicles (< 3 mm) were collected by slicing previously aspirated ovaries. In adult goats, only large follicles (≥ 3 mm) were aspirated. The collection medium of COCs was HEPES-buffered TCM-199 supplemented with 2.2 mg/mL NaHCO_3_, 50 ng/mL gentamycin, 11.1 µg/mL heparin, and in order to avoid spontaneous meiotic resumption 500 µM 3-Isobutyl-1-methylxanthine (IBMX, a phosphodiesterase inhibitor) was added. COCs with homogenous dark cytoplasm and at least two layers of compact cumulus cells were selected.

### Oocyte in vitro maturation

Oocytes from small (< 3 mm) and large (≥ 3 mm) follicles of prepubertal goats and from follicles ≥ 3 mm of adult goats were cultured in pre-IVM, conventional IVM (c-IVM), or CAPA-IVM (pre-IVM followed by a c-IVM) depending on the experiment.

#### Pre-IVM

Pre-IVM medium consisted in TCM-199 with 200 nM CNP, 10 nM 17β-estradiol, 4 mg/mL bovine serum albumin, 0.2 mM sodium pyruvate, 1 mM glutamine, 100 µM cysteamine and 5 µg/mL gentamycin as described by Soto-Heras et al.^18^ The COCs were cultured in pre-IVM medium for 6 h in 100 µL drops (25 – 35 COCs/drop) covered with mineral oil at 38.5 °C in humidified air with 5% CO_2_. A sample of COCs was cultured in pre-IVM medium without CNP and estradiol as a control group.

#### Conventional IVM

c-IVM medium consisted in TCM-199 with 5 µg/mL FSH, 5 µg/mL LH, 3.67 µM 17β-estradiol, 10 ng/mL EGF, 10% (v/v) foetal bovine serum, 0.2 mM sodium pyruvate, 1 mM glutamine, 100 µM cysteamine and 5 µg/mL gentamycin. The COCs were cultured in c-IVM medium for 24 h in 100 µL drops (25 – 35 COCs/drop) covered with mineral oil at 38.5 °C in humidified air with 5% CO_2_.

### Assessment of oocyte nuclear stage

To evaluate meiotic arrest after pre-IVM and nuclear maturation after IVM, the oocyte nuclear stage was assessed. Oocytes were denuded by pipetting and fixed in ethanol with 25 µg/mL Hoechst 33258 overnight at 4 °C. Then, they were covered with Vectashield mounting medium (VectorLabs, CA, USA), flattened with a coverslip, and observed under an epifluorescence microscope (Olympus BX50). Oocyte chromatin was classified following Sui et al.^23^ criteria with a few modifications: GV1 (diffuse filamentous chromatin), GVn (condensed net-like chromatin), GVc (condensed clumped chromatin), GVBD, metaphase I and metaphase II (MII).

### Epidermal growth factor receptor (EGFR) quantification

After IVM, COCs were denuded by pipetting and the EGFR protein was quantified in oocytes and their respective cumulus cells separately.

#### EGFR immunofluorescence

The EGFR in oocytes was quantified by immunofluorescence using a protocol modified from Zhou et al.^24^ Prior to that, we verified the presence of EGFR in goat oocytes by western blotting.^15^

Briefly, denuded oocytes were fixed with 4% paraformaldehyde for 15 min. Oocytes were then permeabilized for 30 min in PBS with 0.25% triton, blocked for 1 h in PBS with 10% normal donkey serum, 3% BSA, and 0.15 M glycine, and incubated with mouse monoclonal anti-EGFR primary antibody (clone 111.6, MA5-13269, Invitrogen, OR, USA) in a 1:20 dilution at 4 °C overnight. Oocytes were then washed 3 times for 10 min in PBS with 0.05% tween and 5% normal donkey serum, and incubated for 2 h at 37 °C with secondary anti-mouse antibody Alexa Fluor 488 conjugated (R37114, Invitrogen) in a 1:1.000 dilution. The DNA was visualized by counterstaining the oocytes with 25 µg/mL Hoechst 33258 for 10 min. Finally, oocytes were covered with Vectashield mounting medium and flattened with a poly-L-lysine treated coverslip, and the slides were stored at −20 °C until analyses the next day. Negative controls were prepared omitting primary antibody.

Immunostained oocytes were observed under an epifluorescence Olympus Fluoview 1000 microscope using an excitation wavelength of 488 nm to visualize EGFR and 405 nm to visualize chromatin. Only MII-oocytes were photographed for EGFR quantification. A single image per oocyte was taken with a camera Hamamatsu ORCA-Flash4.0 LT Plus maintaining the same fluorescence parameters and exposure time. Average fluorescence intensity was measured using ImageJ software (National Institute of Health, MD, USA). The ooplasm area was selected and mean intensity (Total Intensity/Area selected) was quantified.

#### EGFR Western blotting

The EGFR from cumulus cells was analysed by western blotting. After denuding, the cumulus cells from 70–80 COCs were recovered, pelleted, and stored at −20 °C until analyses. Cumulus cell pellets were disrupted, and protein was extracted in RIPA lysis buffer at 4 °C for 1 h. An A431 cell lysate overexpressing EGFR (Servei de Cultius Cel·lulars, Producció d’Anticossos i Citometria, UAB, Spain) was used as a positive control for the presence of EGFR. Protein extractions were loaded into a 7.5% SDS-PAGE gel (Bio-Rad, CA, USA) and transferred to polyvinyl difluoride (PVDF) membranes. For EGFR identification, membranes were blocked in Tris-buffered saline with 0.1% Tween (TBS-T) and 5% BSA for 1 h and incubated overnight at 4 °C with mouse monoclonal anti-EGFR antibody (clone 111.6, MA5-13269, ThermoFisher, USA) diluted 1:50 in 1% BSA-TBS-T. After three 10-min washes, membranes were incubated with anti-mouse secondary antibody horseradish peroxidase conjugated (HRP) (A16017, Invitrogen) diluted 1:2000. Peroxidase activity was revealed using a WesternSure chemiluminescent substrate kit (Li-Cor, Germany) and scanned with C-Digit Blot Scanner and Image Studio Digits software (Li-Cor). Band optical density was quantified by Image Studio Digits software (Li-Cor). To standardize the results, the same membranes were stripped and incubated with a rabbit polyclonal anti-vinculin antibody (926-42215, Li-Cor, 1:1000) followed by an anti-rabbit HRP secondary antibody (926-80011, Li-Cor, 1:5000). EGFR expression was calculated as the ratio between the optical density of the EGFR band and that of vinculin in the same lane. Values for the EGFR expression (measured in arbitrary units) of each replicate were normalized to the value of their corresponding c-IVM group in the same blot, arbitrarily set at 1.

### Measurement of ROS and GSH levels

Intra-oocyte ROS and GSH levels were measured after IVM by staining with 2′,7′-dichlorodihydrofluorescein diacetate (H_2_DCF-DA, Molecular Probes Inc., OR, USA) (adapted from Park et al^25^) or monochlorobimane (MCB) (adapted from Keelan et al.^26^), respectively. Oocytes were denuded and incubated with 10 µM H_2_DCF-DA or 12.5 µM MCB for 15 min in 0.3% BSA-PBS at 38.5 °C. Then, oocytes were washed in 0.1% BSA-PBS, transferred with a 10 µL drop to a slide, and immediately observed under an epifluorescence microscope (Olympus BX50, excitation 460 nm for H_2_DCF-DA or 370 nm for MCB) at a 10x magnification. Images were taken and the average fluorescence intensity per oocyte was measured using ImageJ software and normalized to the average background intensity.

### In vitro embryo production

After IVM, COCs were co-cultured with 4x10^6^ spermatozoa/mL in 100 µL drops of BO-IVF medium (15 COCs/drop; IVF Bioscience, UK) covered with mineral oil at 38.5 °C in humidified air with 5% CO_2_. Prior to IVF, frozen-thawed sperm of two bucks of proven fertility was selected with BoviPure density gradient (Nidacon EVB S.L, Spain) by centrifugation for 15 min at 300 G.

At 20 h post-IVF, presumptive zygotes were gently denuded and cultured in 10 µL drops of BO-IVC medium (8–10 zygotes/drop; IVF Bioscience) covered with Nidoil paraffin oil (Nidacon, Sweden) at 38.5 °C in a humidified atmosphere with 90% N_2_, 5% CO_2_, and 5% O_2_. Five days post-IVF, 5 µL of BO-IVC medium was removed and 5 µL of fresh BO-IVC medium was added to renew nutrients. Cleavage rates were recorded 48 h post-IVF and total embryo and blastocyst rates at 8 days post-IVF.

### Statistical analyses

All data were analysed with two-way ANOVA followed by Tukey’s multiple comparison test using SAS 9.4 software (SAS Inst. Inc., NC, USA). Treatment was set as the fixed factor and replicate as the random variable. Data that did not present a normal distribution and percentage data were square root arcsine transformed prior to ANOVA. Results were considered statistically significant when p < 0.05 and tendencies when p < 0.1.

## Results

### Effect of a 6 h pre-maturation with CNP and estradiol on meiotic arrest

Oocytes from small (< 3 mm) or large (≥ 3 mm) follicles of prepubertal goats were cultured for 6 h in pre-IVM medium either with CNP and estradiol (CNP group) or without them (control group). Oocytes from follicles ≥3 mm of adult goats underwent the same treatment and were used as reference of oocytes with higher competence for comparison. GV rate was assessed to determine meiotic arrest (Table 1). CNP groups from all follicle sizes and puberty statuses maintained a similar GV rate of around 69%, which was higher (p < 0.05) than their respective control groups. The control group of adult goats presented a higher GV rate than both control groups of prepubertal animals.

**Table 1.** Germinal vesicle rate after a 6 h pre-IVM with CNP and estradiol (CNP group) or without them (control group).

Oocyte type	Group	N	GV (%)
Prepubertal small follicles (<3 mm)	CNP	231	155 (69.11 ± 9.85)^a^
	Control	220	63 (28.61 ± 4.21)^c^
Prepubertal large follicles (≥ 3 mm)	CNP	174	112 (67.48 ± 5.59)^a^
	Control	169	45 (30.80 ± 6.77)^c^
Adult follicles (≥ 3 mm)	CNP	119	85 (72.33 ± 3.39)^a^
	Control	120	64 (53.37 ± 4.11)^b^

Data are presented as mean ± S.E.M. Values with different superscript letters differ significantly (p < 0.05). 4–5 replicates were assessed.

**Figure 1.**
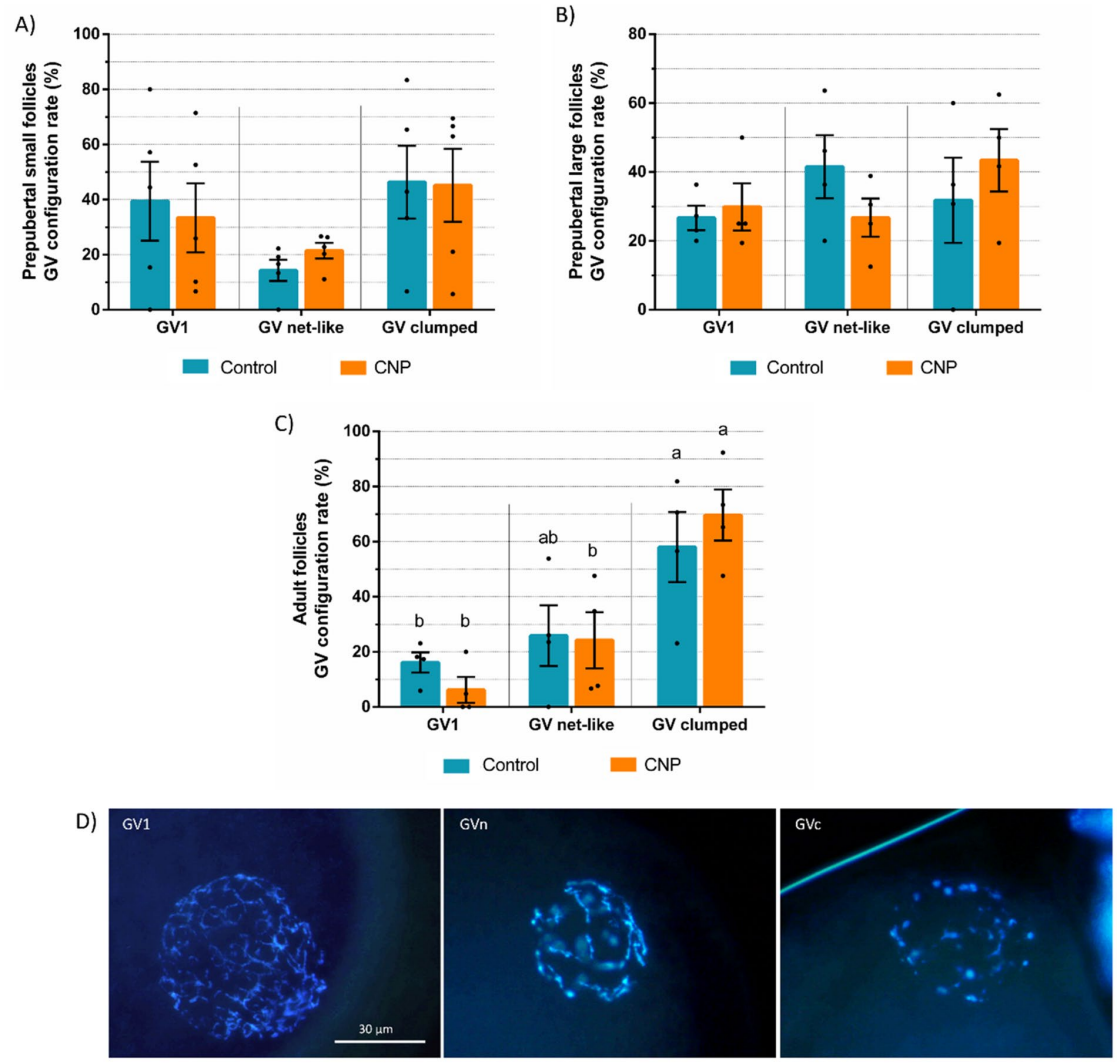
Germinal vesicle chromatin configuration rates of prepubertal goat oocytes from (a) small (< 3 mm) and (b) large (≥ 3 mm) follicles and (c) from follicles ≥ 3 mm of adult females after a 6 h pre-IVM with CNP and estradiol (CNP group) or without them (control group). Each bar represents mean ± S.E.M. Values with different letters differ significantly (p < 0.05). (d) GV configuration representative images on Hoechst-stained oocytes classified as: GV1 (diffuse filamentous chromatin), GVn (condensed net-like chromatin), and GVc (condensed clumped chromatin).

Oocytes that were still in GV were classified according to their chromatin configuration (Fig. 1(d)) as: diffuse filamentous chromatin (GV1), condensed net-like chromatin (GVn), and condensed clumped chromatin (GVc). CNP groups from all follicle sizes and puberty statuses presented similar GV configuration rates to their respective control groups. Prepubertal goat oocytes from small (Fig. 1(a)) and large (Fig. 1(b)) follicles, both CNP and control groups, showed no significant differences between GV1, GVn, and GVc percentages. However, oocytes from adult goats, both CNP and control groups, were predominantly on GVc configuration (Fig. 1(c), p < 0.05).

### Effect of CAPA-IVM on EGF receptor protein expression

**Figure 2.**
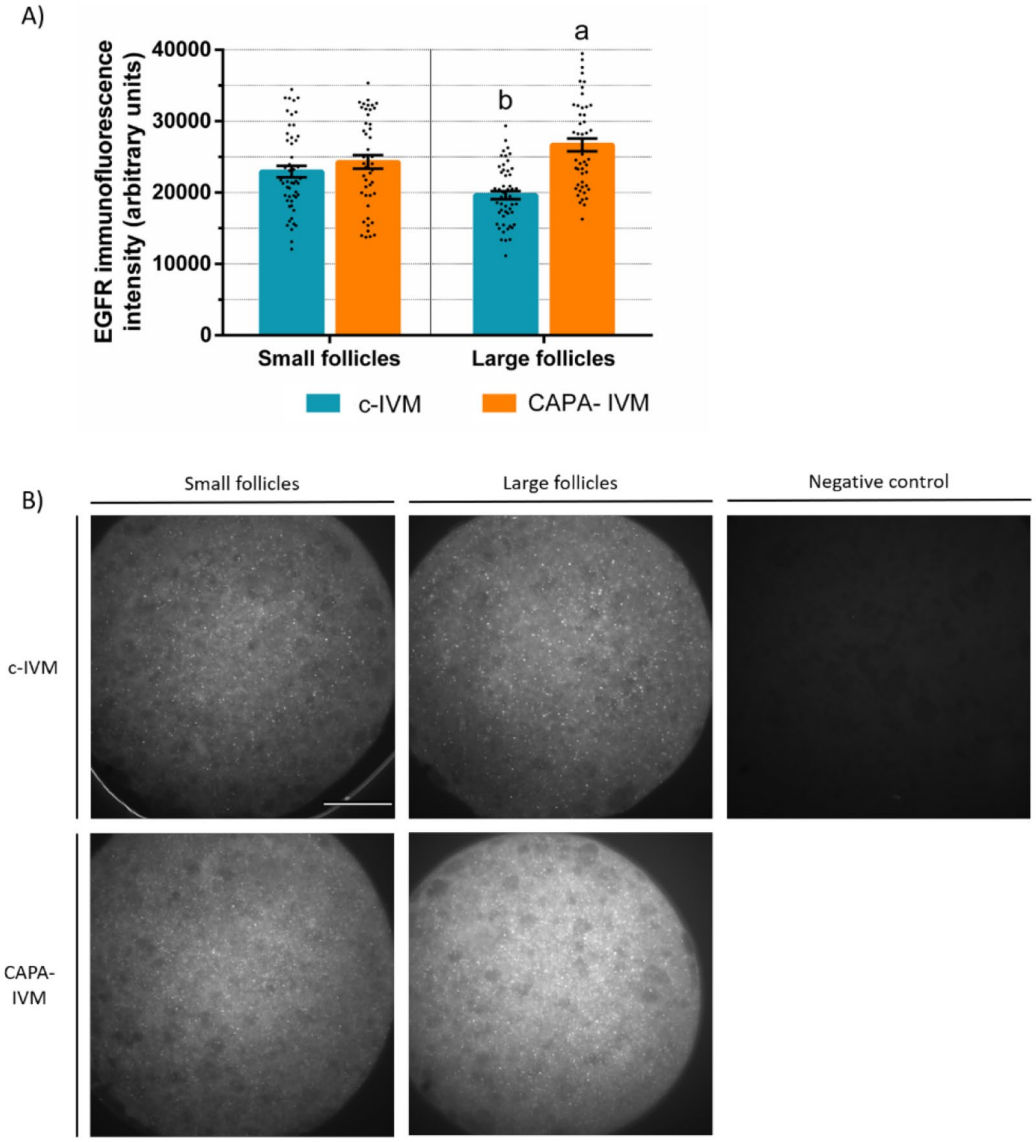
EGFR protein expression in prepubertal goat MII-oocytes from small (< 3 mm) or large (≥ 3 mm) follicles after CAPA-IVM or conventional IVM (c-IVM). (a) fluorescence intensity (mean ± S.E.M) of immunostained oocytes according to follicle diameter. Values with different letters differ significantly (p < 0.05). A total of 47–52 oocytes per group were assessed in 3 replicates. (b) Representative images of immunostained oocytes. Scale bar: 30 µm.

**Figure 3.**
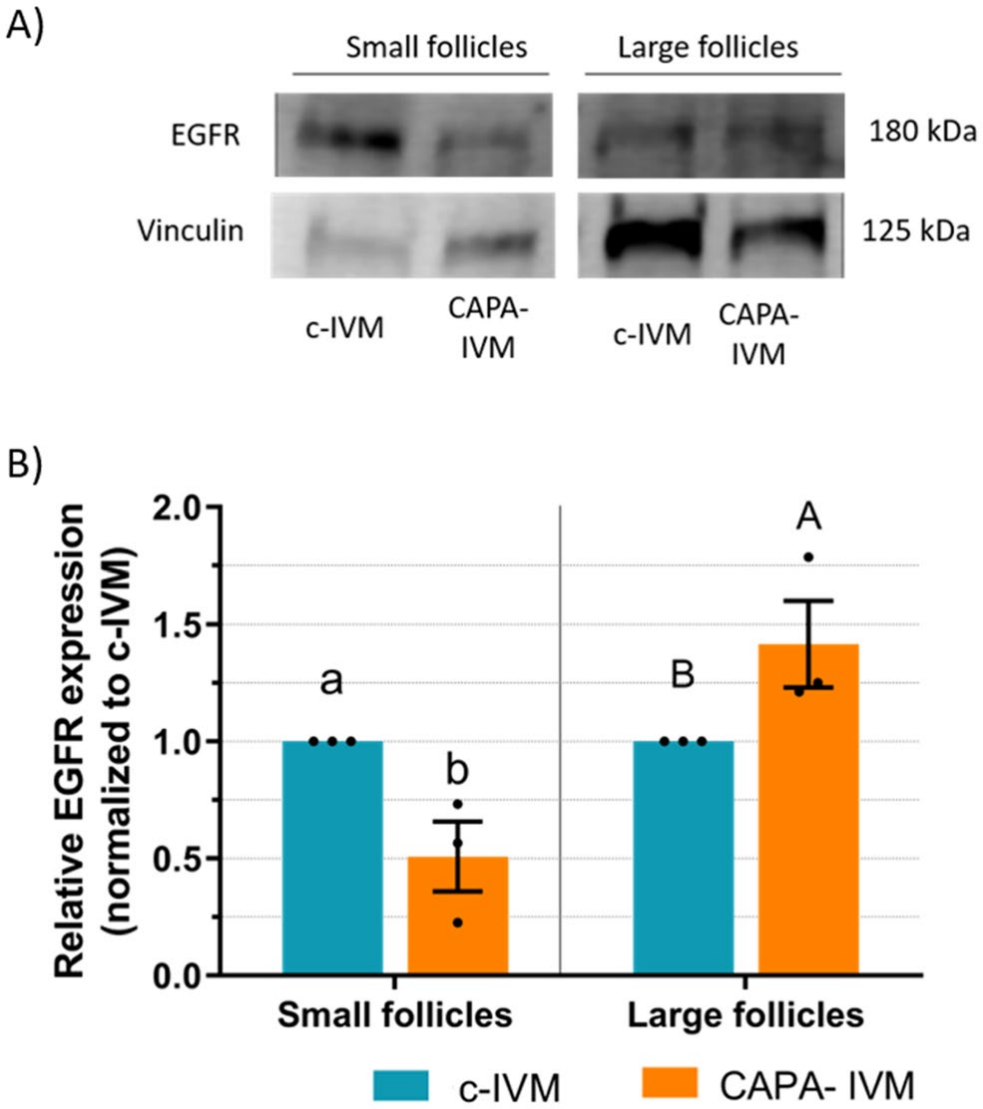
EGFR protein expression in cumulus cells from small (< 3 mm) or large (≥ 3 mm) follicles of prepubertal goats after CAPA-IVM or conventional IVM (c-IVM). (a) Western blot analyses of EGFR expression. EGFR from 70–80 COCs/replicate was quantified by Western blotting and standardized to vinculin protein levels. (b) Relative EGFR expression (mean ± S.E.M). The values of EGFR expression for each replicate were normalized to the value of their corresponding c-IVM group in the same blot, arbitrarily set at 1. Three independent blots were used for relative quantification. Values with different lowercase letters differ significantly (p < 0.05) and with different capital letters show tendency (p < 0.1).

COCs from small (< 3 mm) and large (≥ 3 mm) follicles of prepubertal goats were cultured in CAPA-IVM or c-IVM and the EGFR protein expression was assessed separately in oocytes and their cumulus cells. EGFR expression in MII-oocytes from large follicles was higher after CAPA-IVM compared to c-IVM (p < 0.05, Fig. 2(a)). In MII-oocytes from small follicles, no difference between groups was observed. Regarding EGFR expression in cumulus cells, the ones from large follicles tended to have a higher expression after CAPA-IVM compared to c-IVM (p < 0.1), and the ones from small follicles had a lower expression after CAPA-IVM (p < 0.05, Fig. 3(b)).

### Effect of CAPA-IVM on ROS and GSH levels

**Figure 4.**
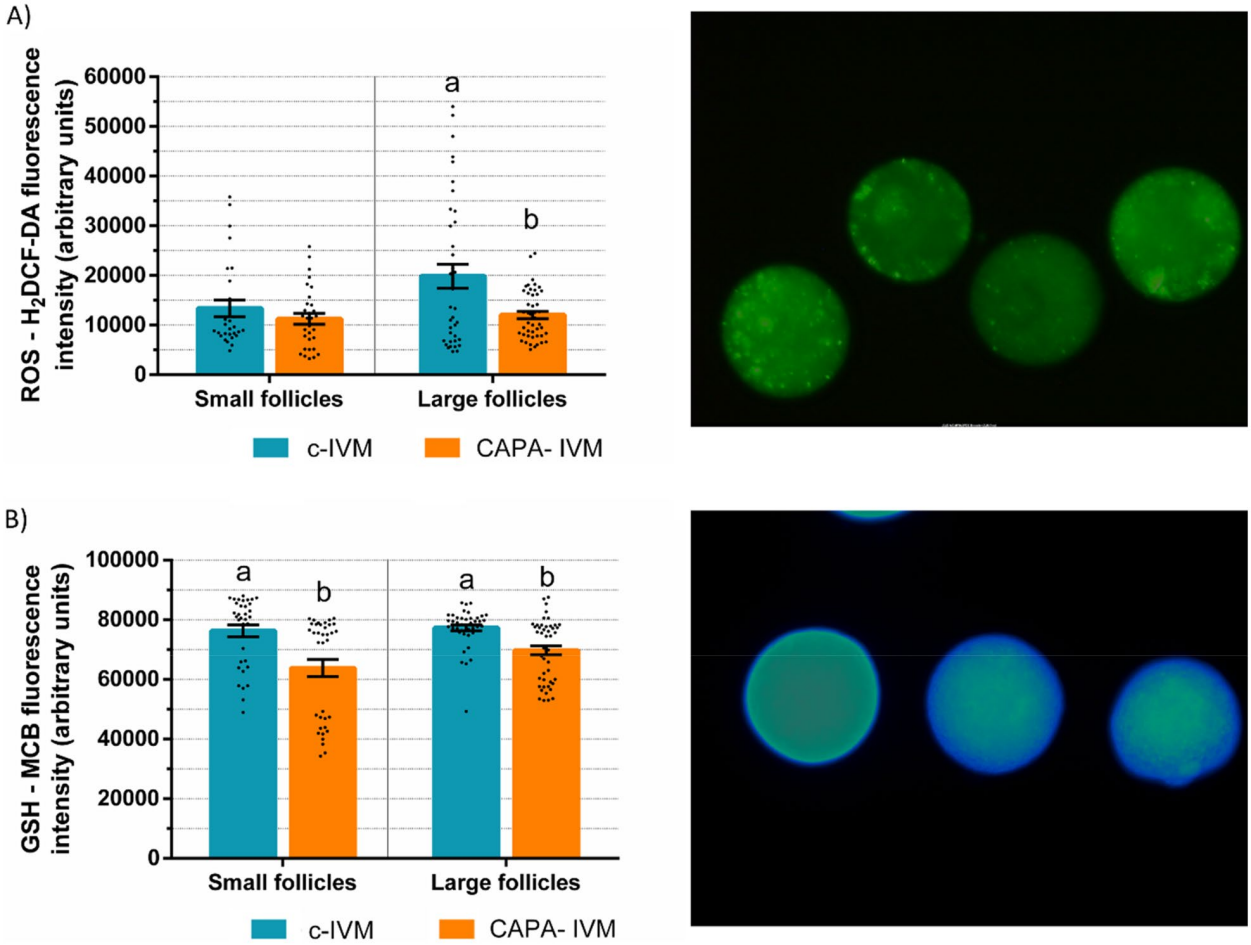
ROS and GSH levels of prepubertal goat oocytes from small (< 3 mm) and large (≥ 3 mm) follicles after CAPA-IVM or conventional IVM (c-IVM). (a) ROS-H_2_DCF-DA and (b) GSH-MCB average fluorescence intensity (mean ± S.E.M) of oocytes and representative images. Values with different superscript letters differ significantly (p < 0.05). A total of 31–47 oocytes per group were assessed in 3–4 replicates.

Prepubertal goat oocytes from small (< 3 mm) and large (≥ 3 mm) follicles were cultured in CAPA-IVM or c-IVM and ROS and GSH levels were assessed. ROS levels in oocytes from large follicles were lower after CAPA-IVM compared to c-IVM (p < 0.05, Fig. 4(a)). In oocytes from small follicles, no difference between groups was observed. GSH levels were lower after CAPA-IVM in oocytes from both small and large follicles (p < 0.05, Fig. 4(b)).

### Effect of CAPA-IVM on nuclear maturation and embryo development

Prepubertal goat oocytes from small (< 3 mm) and large (≥ 3 mm) follicles were cultured in CAPA-IVM or c-IVM, a sample of oocytes were stained for assessing nuclear maturation and the rest were in vitro fertilized and embryo cultured for 8 days. Due to the shortage of animals, adult goat oocytes from follicles ≥ 3 mm were only cultured in c-IVM before nuclear maturation assessment and fertilization for comparison. Prepubertal goat oocytes from small and large follicles provided a similar MII percentage (Table 2), cleavage, embryos >16 cells, and blastocyst rates with no effect of the maturation system used (Table 3). Furthermore, no significant differences were observed in MII percentage, cleavage and >16-cell embryo rates between oocytes from adult and prepubertal females. Regarding blastocyst rates of oocytes from prepubertal goats, only c-IVM oocytes from large follicles did not differ significantly from oocytes of adult goats, and the others were lower (p < 0.05, Table 3).

**Table 2.** Nuclear maturation rate after CAPA-IVM or conventional IVM (c-IVM).

Oocyte type	Group	N	MII (%)
Prepubertal small follicles (<3 mm)	c-IVM	78	57 (71.52 ± 7.67)
	CAPA-IVM	70	56 (77.82 ± 10.10)
Prepubertal large follicles (≥ 3 mm)	c-IVM	105	77 (75.10 ± 9.68)
	CAPA-IVM	91	61 (68.24 ± 3.4)
Adult follicles (≥ 3 mm)	c-IVM	106	96 (90.80 ± 3.93)

Data are presented as mean ± S.E.M. 3 replicates were assessed.

**Table 3.** Cleavage (48 h post-IVF) and embryo development (8 days) after CAPA-IVM or conventional IVM (c-IVM) and IVF.

Oocyte type	Group	N	Cleaved (%)	Embryos >16-cells (%)	Blastocysts (%)
Prepubertal small follicles (<3 mm)	c-IVM	161	90 (59.91 ± 10.24)	27 (17.99 ± 3.61)	6 (3.70 ± 1.13)^a^
	CAPA-IVM	143	76 (57.71 ± 9.33)	30 (20.66 ± 2.61)	5 (2.64 ± 1.73)^a^
Prepubertal large follicles (≥ 3 mm)	c-IVM	109	38 (46.44 ± 15.28)	17 (23.66 ± 10.49)	7 (8.33 ± 4.41)^ab^
	CAPA-IVM	108	57 (56.50 ± 11.55)	17 (16.61 ± 1.90)	3 (2.47 ± 0.88)^a^
Adult follicles (≥ 3 mm)	c-IVM	41	20 (46.42 ± 15.49)	13 (30.39 ± 7.82)	8 (19.02 ± 3.01)^b^

Data are presented as mean ± S.E.M. Values with different superscript letters differ significantly (p < 0.05). 3–5 replicates were assessed.

## Discussion

In this study we assessed the effect of CAPA-IVM on oocyte competence of prepubertal goat oocytes from small (< 3 mm) and large (> 3 mm) follicles. We have observed higher EGFR protein expression in oocytes and cumulus cells, and lower intra-oocyte ROS levels, after CAPA-IVM compared to c-IVM in COCs from large follicles. In COCs from small follicles, these differences were not observed. However, c-IVM and CAPA-IVM produced similar embryo and blastocyst rates in oocytes from both small and large follicles.

In the present study, pre-IVM culture with CNP and estradiol effectively maintained oocytes in meiotic arrest for 6h at a similar GV percentage of around 69% regardless of follicle size and puberty status. Previous studies have already shown that CNP sustains meiotic arrest for 6 h at similar GV rates in prepubertal^18^ and adult goats,^27^^,^^28^ sheep,^29^^,^^30^ cattle^3^^,^^31^^,^^32^ and for 24 h in mouse^33^ and human.^34^ In this study, CNP did not affect the chromatin configuration of GV oocytes compared to the control medium without meiotic inhibitor. Thus, after 6h of pre-IVM, oocytes from prepubertal small and large follicles were evenly distributed among all GV configurations and oocytes from adult goats remained predominantly at the highest stage of condensation (GVc), regardless of treatment. Our observations indicate that although pre-IVM was able to maintain oocytes in meiotic arrest for 6h, it could not stimulate chromatin condensation prior to meiotic resumption. This contrasts with findings of other reports where it was observed that pre-IVM with CNP induced condensed chromatin configurations in cattle,^3^ mouse^33^ and prepubertal goats^18^ alongside an increase in blastocyst development and quality.

Promotion of EGFR signalling improves the quality of low developmental competence oocytes.^35^ At the time of retrieval, we reported higher EGFR protein expression in prepubertal goat oocytes and their cumulus cells from follicles larger than 3 mm compared to follicles smaller than 3 mm.^15^ To determine if CAPA-IVM could enhance the EGF signalling network, in the present study the EGFR protein levels were quantified in oocytes and their cumulus cells after IVM. The expression of EGFR from both cumulus cells and oocytes from large follicles was higher in CAPA-IVM compared to c-IVM. In small follicles, however, the EGFR expression for oocytes was unchanged and it decreased for cumulus cells in CAPA-IVM compared to c-IVM. In cattle, an increased gene expression of EGFR was observed in COCs after a pre-IVM with the cAMP modulators dbcAMP and IBMX, associated with improved developmental competence.^36^ In the pig, the combination of cAMP and the oocyte secreted factors BMP15 and GDF9 increased the phosphorylation of ERK1/2, a downstream effector of EGFR, and the subsequent enhanced blastocyst formation rates were ablated by an EGFR phosphorylation inhibitor.^15^ A pre-IVM with a low dose FSH increased the EGFR functionality, measured as phosphorylation in response to EGF, and subsequent cumulus expansion of porcine COCs.^9^

For determining if CAPA-IVM could enhance the antioxidant defence of the oocyte, intra-oocyte ROS and GSH levels were quantified. ROS-induced oxidative stress impairs maturation and embryo development, and GSH is the main non-enzymatic antioxidant in the oocyte.^37^ The low competence of prepubertal mouse oocytes is related to a higher exposure to ROS due to impaired GSH synthesis.^38^ In the present study, CAPA-IVM decreased ROS levels in oocytes from large follicles, but it had no effect on oocytes from small follicles compared to c-IVM. However, GSH levels were slightly decreased in CAPA-IVM oocytes from small and large follicles. Zhang et al.^32^ observed a decrease of both ROS and GSH levels associated to an increase in blastocyst production in bovine oocytes after CAPA-IVM with CNP. Thus, a possible reason for GSH reduction could be due to it being used for ROS scavenging.^39^ Soto-Heras et al.^31^ observed an increase in mitochondrial activity and blastocyst production after CAPA-IVM but no effect on GSH content in cow oocytes. On the contrary, in our previous study with prepubertal goats CAPA-IVM decreased ROS while it increased GSH and blastocyst yield.^18^

The present study reported that, after c-IVM, oocytes from adult goats had a higher blastocyst rate compared to prepubertal oocytes from small follicles, but there were no differences when compared to prepubertal oocytes from large follicles, in agreement with previous findings obtained in our laboratory.^20^ We have observed in prepubertal goats a positive and direct relationship among follicle diameter, oocyte diameter and embryo developmental competence.^40^^,^^41^ Previous studies in our laboratory showed an increase of blastocyst production after CAPA-IVM,^18^ but contrary to that, in the present study CAPA-IVM did not affect embryo or blastocyst development in oocytes either from small or large follicles even though it was able to reduce ROS content and to stimulate the expression of EGFR in COCs from large follicles. Gilchrist^42^ concluded that small variations in the pre-IVM culture conditions and methodology can have major adverse outcomes on oocyte maturation and embryo yield, even within the same laboratory. While several studies reported a beneficial effect of CAPA-IVM on blastocyst yield,^28^^,^^30^^,^^32^^,^^43^ others reported no effect^3^^,^^27^^,^^44^^,^^45^ or a positive effect only in the presence of other additives in the pre-IVM culture.^31^^,^^33^^,^^46^ In our case, the additional manipulation for sorting oocytes according to follicle size may have caused extra stress and hindered the success of CAPA-IVM. Additionally, Paramio & Izquierdo^47^ reviewed that results for IVEP in goats are highly inconsistent because of the heterogenous and unknown quality of the oocytes. According to Dieci et al.,^22^ the success of CAPA-IVM may be stage-dependent, mainly due to the initial state of differentiation of the COC undergoing the procedure, and it could be further relevant in oocytes with a low inherent competence. These authors observed, after classifying bovine oocytes according to morphological characteristics, that a pre-IVM with cilostamide, an inhibitor of cAMP degradation, improved embryo development in growing oocytes but was detrimental in fully grown oocytes. Similarly, CAPA-IVM with CNP improved the blastocyst yield of oocytes from small follicles but not from medium follicles in sheep (2 to 4 mm and 4 to 6 mm, respectively)^29^ and human (< 6 mm and > 6 mm).^48^ In pigs, CAPA-IVM increased blastocyst development in medium follicles (3 to 6 mm) but it was detrimental in large follicles (6 to 8 mm).^49^ In contrast, when bovine oocytes were recovered from very small follicles (< 3 mm), no beneficial effect of CAPA-IVM was observed while in medium sized follicles (3 to 8 mm) it induced an increase in blastocyst rates.^50^ These authors hypothesized that a short 6h period of meiotic arrest prior to IVM was insufficient to adequately support cytoplasmic maturation of oocytes derived from very small bovine follicles and that signalling networks in these COCs might be insufficient to promote oocyte cytoplasmic maturation.

Overall, our results suggest that CAPA-IVM with CNP and estradiol enhanced EGFR expression for EGF peptide signalling and antioxidant capacity in oocytes from follicles larger than 3 mm but this effect was not translated to an increase in blastocyst development. However, oocytes from follicles smaller than 3 mm were too immature to benefit from a short 6h pre-IVM culture.

## Data Availability

The dataset supporting the conclusions of this article is available in the CORA.RDR repository, https://doi.org/10.34810/data1389
